# Electroencephalography during general anaesthesia differs between term-born and premature-born children

**DOI:** 10.1016/j.clinph.2015.10.041

**Published:** 2016-02

**Authors:** Ravi Poorun, Caroline Hartley, Sezgi Goksan, Alan Worley, Stewart Boyd, Laura Cornelissen, Charles Berde, Richard Rogers, Tariq Ali, Rebeccah Slater

**Affiliations:** a Nuffield Department of Clinical Neurosciences, University of Oxford, John Radcliffe Hospital, Oxford OX3 9DU, UK; b Department of Paediatrics, University of Oxford, John Radcliffe Hospital, Oxford OX3 9DU, UK; c Department of Clinical Neurophysiology, Great Ormond Street Hospital for Children, London WC1N 3JH, UK; d Department of Anesthesiology, Perioperative and Pain Medicine, Boston Children’s Hospital, Boston, MA 02115, USA; e Nuffield Department of Anaesthetics, John Radcliffe Hospital, Oxford OX3 9DU, UK

**Keywords:** EEG, Nociception, Preterm, Anaesthesia

## Abstract

**Objectives:**

Premature birth is associated with a wide range of complications in later life, including structural and functional neurological abnormalities and altered pain sensitivity. We investigated whether during anaesthesia premature-born children display different patterns of background EEG activity and exhibit increased responses to nociceptive stimuli.

**Methods:**

We examined background EEG and time-locked responses to clinical cannulation in 45 children (mean age (±SD) at study: 4.9 (± 3.0) years) under sevoflurane monoanaesthesia maintained at a steady-state end-tidal concentration of 2.5%. 15 were born prematurely (mean gestational age at birth: 29.2 ± 3.9 weeks) and 30 were age-matched term-born children.

**Results:**

Background levels of alpha and beta power were significantly lower in the premature-born children compared to term-born controls (*p* = 0.048). Clinical cannulation evoked a significant increase in delta activity (*p* = 0.032), which was not significantly different between the two groups (*p* = 0.44).

**Conclusions:**

The results indicate that whilst under anaesthesia premature-born children display different patterns of background brain activity compared to term-born children.

**Significance:**

As electrophysiological techniques are increasingly used by anaesthetists to gauge anaesthetic depth, differences in background levels of electrophysiological brain activity between premature and term-born children may be relevant when considering titration of anaesthetic dose.

## Highlights

• Noxious stimulation during anaesthesia evokes a significant increase in delta activity that does not differ between term-born and premature-born children.
• Background EEG activity recorded during anaesthesia is different in premature-born and term-born children.
• EEG-derived measures that can be used to titrate anaesthetic depth may be influenced by premature birth.

## 1 Introduction

Optimal titration of anaesthetic dose is essential to achieve effective analgesia, unconsciousness and immobility, and to avoid the dangers of both under and over dosing. Anaesthetists primarily rely on autonomic and behavioural responses to optimise levels of anaesthesia and provide appropriate analgesia. However, more recently electrophysiological measures of brain activity (such as spectral measures, entropy and bispectral activity) have been used to gauge anaesthetic depth (Davidson, 2007, Palanca et al., 2009, Ni Mhuircheartaigh et al., 2013, Marchant et al., 2014, McKeever et al., 2014, Sury et al., 2014) and to identify changes in brain activity evoked by nociceptive stimuli (Kochs et al., 1994, Bischoff et al., 1996, Hagihira et al., 2004, Morimoto et al., 2005, Sleigh et al., 2010, Hartley et al., 2014). Indeed, autonomic and motor responses alone are insufficient to assess analgesia in the anaesthetised patient, since these responses may reflect spinal and brainstem actions, rather than specific cortical processing of nociceptive stimuli, which is a prerequisite for the experience of pain.

However, one challenge of measuring electrophysiological brain activity in anaesthetised patients is that different patient groups can respond differently to the anaesthesia. For example, children with intellectual impairments or cerebral palsy appear to reach a deeper level of anaesthesia when administered with the same dose of anaesthetic compared to children considered to be cognitively normal (Choudhry and Brenn, 2002, Valkenburg et al., 2009, Edry et al., 2011). Hence, there is a strong clinical rationale for developing electrophysiological measures and to optimise them for different patient groups. As premature birth is associated with long-term structural and functional neurological abnormalities (Anjari et al., 2007, Nosarti et al., 2008, Johnson et al., 2009b, Doesburg et al., 2011), it is plausible that during anaesthesia premature-born children may exhibit altered patterns of electrophysiological brain activity compared to term-born children. This may be particularly important during anaesthetic practice as premature-born children may undergo multiple operations during critical periods of brain development.

During wakefulness, premature-born children show reduced alpha band power in their background EEG activity compared with term-born children (Doesburg et al., 2011, Doesburg et al., 2013b), and a decrease in alpha and beta power has been observed in premature-born adults (Miskovic et al., 2009). If monitoring electrophysiological measures of brain activity is to be clinically useful during paediatric anaesthesia, then understanding whether these differences in brain activity also occur under anaesthesia is beneficial. Furthermore, evidence suggests that infants who experience a high burden of pain in early life may be more sensitive to subsequent pain exposure (Taddio et al., 1997, Taddio et al., 2002, Peters et al., 2005, Hermann et al., 2006, Hohmeister et al., 2009, Hohmeister et al., 2010, Slater et al., 2010). Indeed, during prematurity neural pathways are still undergoing rapid maturation and nociceptive input during this period can alter the normal course of development, which can lead to long-term changes in nociceptive sensitivity. Peters and colleagues reported an increase in perioperative analgesic requirement and higher post-operative pain to subsequent surgery in children who had undergone early life surgery (Peters et al., 2005). Similarly, premature-born children may also have increased responses to noxious stimulation during surgery.

We have recently demonstrated that children exhibit an increase in delta band activity in response to noxious and innocuous stimulation whilst under anaesthesia (Hartley et al., 2014). The aim of this prospective study was to determine whether premature-born children exhibit altered patterns of background brain activity compared to term-born children and to establish whether their electrophysiological brain responses following nociceptive stimulation are altered. We hypothesised (i) that premature-born children would have a greater increase in evoked delta activity in response to clinical cannulation than term-born children, and (ii) that anaesthetised premature-born children would have reduced alpha and beta power in their background EEG activity.

## 2 Methods

### 2.1 Subjects

Ethical approval was granted by the Oxford Research Ethics Committee of the National Research Ethics Service. Informed written parental consent and, where appropriate, the child’s assent were obtained prior to each study. The study conformed to the standards set by the Declaration of Helsinki and Good Clinical Practice guidelines.

Forty-five children aged 1–12 years receiving an elective operation or investigation (MRI) under general anaesthesia were recruited from the John Radcliffe Children’s Hospital, Oxford between July 2012 and February 2014. Thirty children were recruited as part of the main study, with 15 premature-born children (mean gestational age at birth: 29.2 weeks, range 23–34 weeks, mean age at study: 5.2 years, range 15–150 months) and 15 aged-matched term-born children (mean age at study: 5.2 years, range 18–153 months). As the sample size is relatively small, and there is a higher prevalence of term-born children, we wanted to be sure that the mean values and standard errors that we reported in the term-born children were reproducible in an independent sample. Therefore, a separate cohort of 15 aged-matched term-born children (mean age at study: 4.4 years, range 14–100 months) were recruited for secondary comparison. 39 of the 45 children included in this analysis were included in a previous study that initially characterised the electrophysiological brain response following innocuous and noxious stimulation in anaesthetised children (Hartley et al., 2014).

Children were eligible for inclusion in the study if a gaseous induction of anaesthesia was required. Children were excluded if they received premedication before anaesthetic induction, if they required emergency care, or if they required an intravenous (IV) anaesthetic induction. Term-born children were additionally excluded from the study if they had central nervous system disease or developmental delay. As prematurity leads to a number of developmental problems, premature-born children were not excluded on these grounds – instead the study was designed to investigate the range of premature-born children seen in the clinical setting (see Supplementary Table S1 for further subject details). However, given this pragmatic approach, a number of the premature-born children had overt neurological impairment. In part of the analysis the premature-born children were split into two groups according to whether they had been clinically diagnosed with a neurological impairment, as recorded in medical notes (see Supplementary Table S1).

### 2.2 Anaesthetic procedures

**Fig. 1:**
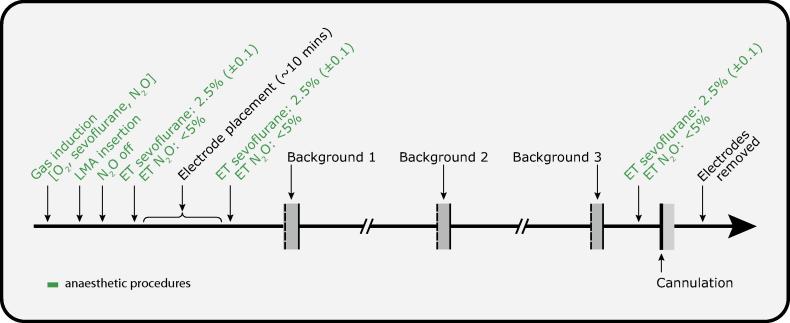
Experimental protocol. Anaesthetic procedures are shown in green and experimental procedures in black. All subjects had a gas induction with sevoflurane and nitrous oxide (N_2_O), after which a laryngeal mask airway (LMA) was inserted. The end-tidal (ET) sevoflurane was set to 2.5% and N_2_O was <5% during the experimental recording. Three background periods were taken across a recording period of approximately 2 min prior to cannulation. (For interpretation of the references to colour in this figure legend, the reader is referred to the web version of this article.)

Anaesthetic procedures were as described previously (Hartley et al., 2014) and are outlined in Fig. 1. Gaseous induction of anaesthesia was performed using sevoflurane (Baxter, UK), oxygen and nitrous oxide following routine anaesthetic practice. Once stable a laryngeal mask airway was inserted, sevoflurane was reduced to achieve an end-tidal concentration of 2.5% and nitrous oxide was turned off. The time between the induction of anaesthesia and starting the recordings was at least 10 min (the time for electrode placement), at which point end-tidal nitrous oxide levels were confirmed to be less than 5% and sevoflurane levels stabilised to an end-tidal concentration of 2.5%, equivalent to 1 minimum alveolar concentration (MAC) of sevoflurane across the participant age range (Lerman et al., 1994).

### 2.3 Experimental recording techniques

Recording techniques were as described previously (Hartley et al., 2014). Immediately after anaesthetic induction eight EEG recording electrodes (Ambu Neuroline disposable Ag/AgCl cup electrodes) were positioned on the scalp at Cz, CPz, C3, C4, Fz, FCz, T3 and T4 according to the modified international 10–20 system. Reference and ground electrodes were placed at Fpz and the forehead, respectively. A limited electrode montage was used because it was a priority that electrode placement were completed in the shortest time possible. This enabled the cannulation to be performed soon after induction so that venous access could be gained and ensured that the time in which the children were anaesthetised was not unduly increased. EEG conductive paste (Elefix EEG paste, Nihon Kohden) was used to optimise contact with the scalp. All impedances were kept below 5 kΩ by rubbing the skin with EEG preparation gel (NuPrep gel, D.O. Weaver and Co.) prior to electrode placement. Electrophysiological activity was acquired with the SynAmps RT 64-channel headbox and amplifiers (Compumedics Neuroscan), with a bandwidth from DC – 400 Hz and a sampling rate of 2 kHz. CURRYscan7 neuroimaging suite (Compumedics Neuroscan) was used to record the activity.

### 2.4 Stimulation

Clinically-required cannulation was time-locked to electrophysiological recordings by means of a high-speed camera (Firefly MV, Point Grey Research Inc.) that was directly linked to the recordings at the time of acquisition (Hartley et al., 2014). Video recordings were captured at 220 frames per second. The exact point of cannulation was then marked on the electrophysiological recording after the experiment, defined as the point where the cannula first made contact with the surface of the skin (Hartley et al., 2014). Cannulation was performed on the dorsum of the hand, which was selected by the anaesthetist (in 73% of studies the left hand was selected).

### 2.5 EEG analysis

The EEG trace was segmented into epochs of 5 s before and 5 s after the cannulation. Each epoch was baseline corrected, low pass filtered at 70 Hz and notch filtered at 50 Hz. The power spectrum was calculated in MATLAB (version R2013a, MathWorks) using the fast Fourier transform. The power (quantified as the absolute power) in the delta (0–3 Hz), theta (3–8 Hz), alpha (8–12 Hz) and beta (12–30 Hz) frequency bands were defined as the total power within these frequency ranges.

Three 5-s epochs of background EEG activity were analysed at the beginning, middle and end of an approximately 2-min recording taken prior to cannulation. During this 2-min period tactile and experimental noxious stimuli were applied (see previous study (Hartley et al., 2014)) but not included in this analysis. Background periods were selected during periods of EEG activity where no stimulation was performed. In the period immediately prior to the cannulation the upper limb was prepared for cannulation. Therefore, EEG activity in this period included activity associated with performing the cannulation and was not used in the background analysis. The change in band power, from background, evoked by cannulation was compared to the change in band power between two background periods (see Fig. 1), at Cz and CPz (Hartley et al., 2014).

### 2.6 Statistical analysis

Statistical analysis was carried out using R (The R Project for Statistical Computing). Assessment of QQ plots showed that delta, theta, alpha and beta power, as well as the change in delta power, were all non-normally distributed. The four band powers were normalised prior to statistical testing by taking the logarithm. The change in delta power (i.e. the difference in delta band power in response to cannulation with background activity) could not be normalised and so non-parametric tests were used. The change in delta power in response to cannulation from background levels (with both the premature-born and term-born groups pooled together) was compared with the change between two background periods using a permutation test with subject age and EEG channel as additional factors. The change in delta power in response to cannulation was compared between the two aged-matched groups using a permutation test, with subject group (premature-born or term-born) and EEG channel as within subject factors. For each frequency band (i.e. delta, theta, alpha and beta), the background EEG was compared between the age-matched subject groups using a three-way ANOVA (with subject group, background period and EEG channel all within subject factors). Holm’s method was used to correct for multiple comparisons across the four band powers.

## 3 Results

### 3.1 Noxious cannulation evoked a significant increase in delta power that is not significantly different between premature-born and term-born children

**Fig. 2:**
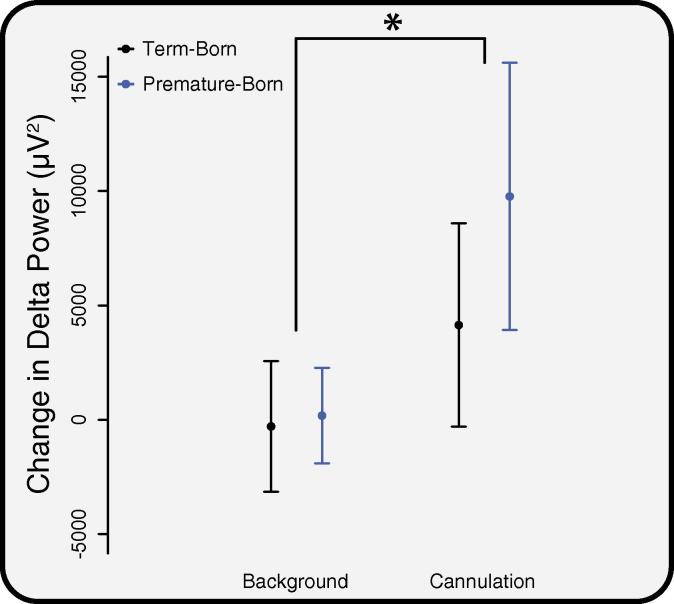
The change in delta band power in response to cannulation. The change in delta band power from cannulation to background activity (‘cannulation’) is compared with the change in background activity between two background periods (‘background’). There were no significant differences in the change between the premature-born (blue) and term-born (black) groups, though a significant increase was observed between background and cannulation (*: *p* < 0.05). Error bars indicate standard error of the mean. (For interpretation of the references to colour in this figure legend, the reader is referred to the web version of this article.)

Noxious cannulation evoked a significant increase in delta power in both term-born and premature-born children (*p* = 0.032, permutation test, with no effect of channel or age). However, the increase in delta activity was not significantly different between the term-born and premature-born groups (Fig. 2, *p* = 0.44, permutation test). The average background delta power in the term-born children was 1.6 ± 0.2 × 10^4^ μV^2^ (mean ± standard error of the mean (SEM)) and in the premature-born children was 1.4 ± 0.2 × 10^4^ μV^2^. The average delta power following cannulation was 2.0 ± 0.3 × 10^4^ μV^2^ in the term-born children and 2.5 ± 0.4 × 10^4^ μV^2^ in the premature-born children. There was no affect of age on the change in delta power (Supplementary Fig. S1A).

### 3.2 Background alpha and beta power is significantly lower in anaesthetised premature-born children compared to age-matched term-born children

**Fig. 3:**
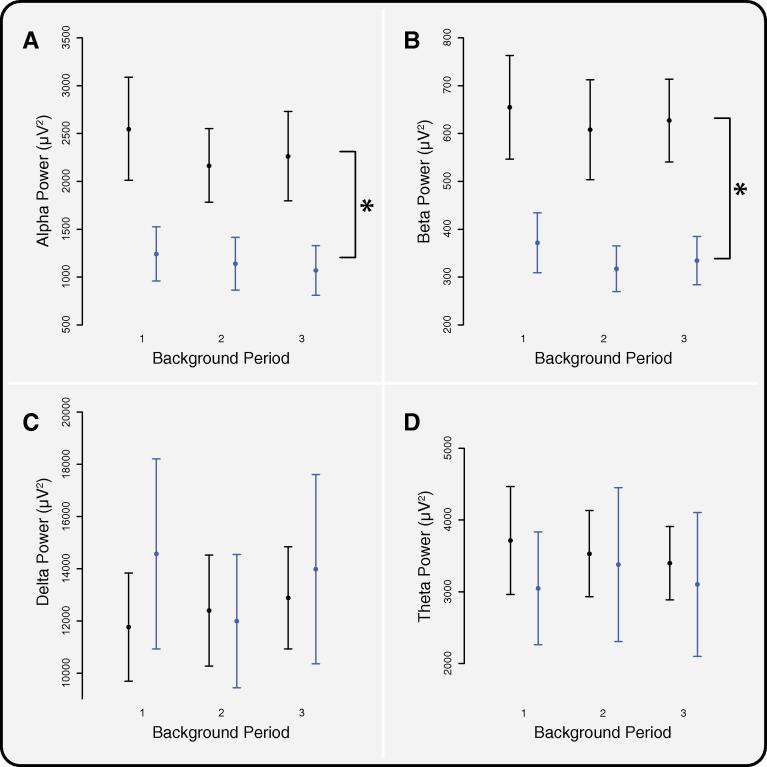
Comparison of background activity between premature-born and term-born children. (A) Alpha, (B) beta, (C) delta, and (D) theta band powers across the three background periods for the term-born (black) and premature-born (blue) groups. (*: *p* < 0.05 indicates significant differences between the two groups across all background periods, *p*-values were corrected for multiple comparisons using Holm’s method, across the 4 frequency bands.) Error bars indicate standard error of the mean. (For interpretation of the references to colour in this figure legend, the reader is referred to the web version of this article.)

The average background power spectrum for the premature-born and term-born children is shown in Supplementary Fig. S2. The background delta power (*p* = 0.66; three-way ANOVA) and theta power (*p* = 0.28) was not significantly different between the two groups of children (Fig. 3). However, the background alpha power (*p* = 0.048) and beta power (*p* = 0.048, *p*-values corrected for multiple comparisons across the four frequency bands) were significantly lower in the premature-born children compared with the term-born children (average power – premature-born children: alpha = 1150 ± 272 μV^2^, beta = 341 ± 54 μV^2^, term-born children: alpha = 2326 ± 468 μV^2^, beta = 630 ± 100 μV^2^, mean ± SEM, Fig. 3). Pooling both groups, there was no affect of age on background alpha or beta power (*p* = 0.60 and 0.70, respectively, Supplementary Fig. S1B and C).

The decrease in alpha and beta power in the premature-born children was observed across all three background periods (see Fig. 3) and both the alpha and beta power remained stable across these periods (*p* = 0.36 and 0.16, respectively; three-way ANOVA). The alpha and beta power in the premature-born children were lower across all EEG channels (compared with the term-born children), however, there was a significant effect of channel (*p* < 0.001; three-way ANOVA) with the greatest difference in band power observed at FCz, Fz and Cz (Supplementary Fig. S3). To confirm the validity and robustness of this observation, the alpha and beta power in the term-born children was compared to an independent sample of age-matched term-born children (*n* = 15). The alpha and beta power was consistent between the two groups of term-born children (Supplementary Fig. S4, *p* > 0.05), and the premature-born children had significantly lower alpha and beta power compared with the second group of term-born children (*p* = 0.042 and 0.008, respectively). This indicates that the decrease in alpha and beta power in the premature-born children relates to the group’s prematurity rather than general subject variability.

### 3.3 Background EEG and brain pathology

**Fig. 4:**
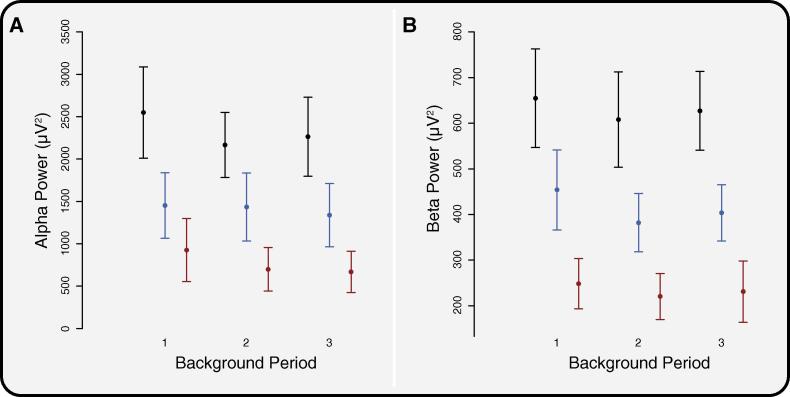
Comparison of children with and without neurological impairment. (A) Alpha and (B) beta power across the three background periods in the term-born children (black), compared with the premature-born children without neurological impairment (blue) and the premature-born children with neurological impairment (red). Error bars indicate standard error of the mean. (For interpretation of the references to colour in this figure legend, the reader is referred to the web version of this article.)

In this study we considered a clinically-representative population of premature-born children with a range of pathologies, including overt neurological impairment (see Supplementary Table S1). The premature-born children with overt neurological impairment (*n* = 6) had lower average levels of background alpha and beta power (764.3 ± 230.0 μV^2^ and 233.2 ± 55.2 μV^2^, respectively, mean ± SEM) compared to the premature-born children without overt neurological impairment (*n* = 9, average alpha: 1408.4 ± 373.1 μV^2^, average beta: 413.2 ± 66.0 μV^2^). However, the average alpha and beta power in the premature-born children without overt neurological impairment were still lower than the term-born children (Fig. 4). Thus, whilst the reduction in alpha and beta power in the premature-born children is related to neurological impairment, it is also a feature of the whole group.

## 4 Discussion

In our previous study we demonstrated that clinical noxious cannulation evoked a significant increase in delta activity (Hartley et al., 2014). A similar approach has been used in this study to quantify the differences in the brain’s electrical activity between aged-matched premature-born and term-born children under sevoflurane anaesthesia. Contrary to our hypothesis, the activity evoked by cannulation was not significantly different between the two groups of children, suggesting that nociceptive processing in anaesthetised children is not significantly altered by prematurity. Nevertheless, when considering background EEG activity, as hypothesised, we observed stark differences between the premature-born and term-born children.

### 4.1 Thalamocortical interactions may underlie the alterations in alpha and beta power in premature-born children

Previous research in awake premature-born children has demonstrated a reduction in alpha power (Doesburg et al., 2011) and an increase in the gamma/alpha ratio (Doesburg et al., 2013a). A decrease in alpha and beta power has also been observed in premature-born adults (Miskovic et al., 2009). These spectral changes are correlated with an increased likelihood for premature-born infants to experience later cognitive deficits (Doesburg et al., 2013a, Doesburg et al., 2013b). Alpha and beta power are also reduced in pathologies (Hughes and Crunelli, 2005), including Alzheimer’s disease, in which EEG spectral measures correlate with scores of cognitive deterioration (Jeong, 2004). As thalamocortical interactions are thought to underlie alpha rhythm generation it has been suggested that these changes in spectral power relate to alterations in thalamocortical connectivity (Hughes and Crunelli, 2005). Even without overt neurological sequelae, premature-born children may have structural and functional white matter alterations (Anjari et al., 2007, Vinall et al., 2014). Thus, it has been suggested that changes in thalamocortical circuits also leads to alterations in spectral power in awake premature-born children (Doesburg et al., 2011). Whilst a limitation of this study is that EEG activity was not recorded before induction of anaesthesia, we demonstrate that the spectral differences observed between term and premature-born children whilst awake (Miskovic et al., 2009, Doesburg et al., 2011, Doesburg et al., 2013b), also occur under anaesthesia. Whether anaesthesia exacerbates or reduces these differences should be investigated in a future study. However, as thalamocortical feedback loops are thought to drive alpha activity under anaesthesia (Ching et al., 2010), it seems plausible that alterations in thalamocortical circuits in premature-born children also gives rise to the reduction in alpha power observed here in premature-born children under anaesthesia.

### 4.2 Clinical monitors of anaesthetic depth may be altered in premature-born children

In recent years there has been a drive towards the use of EEG monitoring to measure depth of anaesthesia (Ni Mhuircheartaigh et al., 2013, Marchant et al., 2014, Sury et al., 2014). There is much controversy over the use of such monitors in adults, and the validity is even more problematic in children (Davidson, 2007, Palanca et al., 2009, McKeever et al., 2014). Indeed, the need for age-adjusted measures for paediatric anaesthetic depth monitoring has been demonstrated (Cornelissen et al., 2015). It is too early to make clinical recommendations regarding endpoints for titration of anaesthetic depth in premature-born children based on the results shown here. However, given the background EEG differences, it is worth noting that current depth of anaesthesia monitors may be less accurate in premature-born children compared with their term-born counterparts. Understanding how relevant clinical factors (such as premature birth) affect measures of anaesthetic depth would be essential if these electrophysiological measures are to be successfully utilised in the clinical setting.

Electrophysiological measures used to assess anaesthetic depth have been reported to be lower in children with intellectual impairments (Valkenburg et al., 2009, Edry et al., 2011). Children with cerebral palsy require less propofol during induction to reach steady state anaesthetic depth monitor values compared with controls (Saricaoglu et al., 2005), and have lower values at set anaesthetic levels (Choudhry and Brenn, 2002). It therefore seems likely that premature-born children with neurological pathology will have lower anaesthetic depth monitor values at the same level of anaesthetic. Indeed, in this study the children with the lowest alpha and beta powers were those with overt neurological impairment.

We took a pragmatic approach to the recruitment of premature-born children, aiming to study the spectrum of premature-born children seen in the clinical setting. The premature-born children were born at a wide range of gestations from 23 to 34 weeks, and 6 out of 15 had overt neurological impairment. Whilst the greatest reduction in alpha and beta band power (compared with term-born children) was observed in the premature-born children with overt neurological impairment, a reduction in power was also seen in the premature-born children without overt neurological impairment. Even without overt pathologies, premature-born children are at higher risk of cognitive impairment (Grunau et al., 2002, Johnson et al., 2009a, Johnson et al., 2009b). Future studies should therefore investigate background EEG measures under anaesthesia compared with neuropsychological indices. We did not find a relationship between age and background alpha and beta power, or the magnitude of the evoked delta response to cannulation. Whilst the age range was relatively wide (1–12 years), 57% of the participants were between 3 to 6 years. Given the relatively small sample size, a future study is needed to assess any age related changes, and also the affect of the degree of prematurity.

### 4.3 Responses to cannulation under anaesthesia are not altered by premature birth

Children who are born prematurely often experience multiple painful procedures as part of their essential medical care (Carbajal et al., 2008) and previous studies in awake premature-born children have demonstrated that exposure to pain in early infancy can lead to altered subsequent pain experience (Hermann et al., 2006, Hohmeister et al., 2010, Slater et al., 2010). However in this study, and contrary to our hypothesis, no difference in the response to cannulation was observed between the premature-born and term-born children during anaesthesia.

Pain is a subjective experience, influenced by many factors such as memories, social context, attention and mood (Tracey and Mantyh, 2007). It is therefore plausible that heightened pain sensitivity, which has been reported in awake premature-born children, may be influenced by environmental or psychological factors that are not relevant in the anaesthetised child. For example, it is reported that premature-born children have a heightened level of pain catastrophising (Hohmeister et al., 2009), which may contribute to an altered pain experience in the awake child but would not be relevant to nociceptive processing in the anaesthetised child. As we did not observe differences here between anaesthetised premature-born and term-born children in response to cannulation, this leads to the possibility that altered pain sensitivity observed in awake premature and term-born individuals may be predominantly related to the conscious processing of the stimulus. Nevertheless, this is not the only explanation, and it is also plausible that if the premature-born children were at a different depth of anaesthesia compared with the term-born children (which might be inferred from the differences in background alpha and beta power), that this would influence their response to noxious stimulation and potentially lead to no differences being observed between the groups. In this study, the observed differences cannot be attributed to movement or changes in autonomic activity, as cannulation does not lead to concomitant changes in spinal reflex withdrawal or heart rate (Hartley et al., 2014).

### 4.4 Conclusions

We demonstrated that premature-born children have decreased alpha and beta power compared with term-born controls whilst under sevoflurane anaesthesia, and that both groups show an increase in delta power in response to cannulation. Moreover, we show that when the same level of anaesthetic is given, premature-born and term-born children display similar responses to nociceptive stimulation. This suggests that the altered sensitivity to nociceptive input reported in awake premature-born children may not occur when noxious stimuli are applied to the unconscious child. Differences in background brain activity in premature-born children may be relevant when considering titration of anaesthetic dose.

## Appendix A Supplementary data

Supplementary Fig. S1 Relationship between patterns of brain activity with age. (A) The change in delta power in response to cannulation, (B) background alpha power, and (C) background beta power, plotted against age at study for term-born (black) and premature-born (blue) children. Each point represents the average EEG activity across channels for each child.

Supplementary Fig. S2 Average power spectra in the background EEG. Power spectra, averaged across all channels and all children, in the background period for the term-born (black) and premature-born (blue) children. For the analysis the power was calculated in the delta (0–3 Hz, red), theta (3–8 Hz, light blue), alpha (8–12 Hz, yellow), and beta (12–30 Hz, green) bands.

Supplementary Fig. S3 Comparison of band power with EEG electrode. (A) Alpha and (B) beta power in the first background period for each EEG electrode, compared between term-born (black) and premature-born (blue) children. Error bars indicate standard error of the mean.

Supplementary Fig. S4 Comparison of band power in two groups of term-born children. (A) Alpha and (B) beta power across the three background periods in the group of term-born children studied in the main text (black) compared with a second group of term-born children (green). There were no significant differences between the two groups. Error bars indicate standard error of the mean.

Supplementary Table S1 Subject age and procedure details. Children are listed according to age, with age-matching indicated by each row of the table. Term-born children (blue rows) are divided according to whether they were in the main study (left hand side) or group 2 (right hand side). Details highlighted in red for the premature-born children (green rows) indicate those children with neurological impairment.
