# Supplementary figures and images for: Electroencephalography during general anaesthesia differs between term-born and premature-born children
Source: Clin Neurophysiol. 2016 Feb;127(2):1216–22. doi: 10.1016/j.clinph.2015.10.041 (PMC4725254; doi:10.1016/j.clinph.2015.10.041)

A

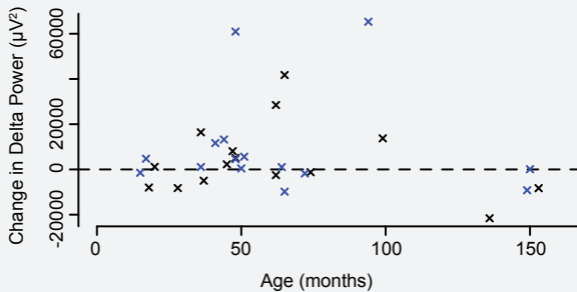

B

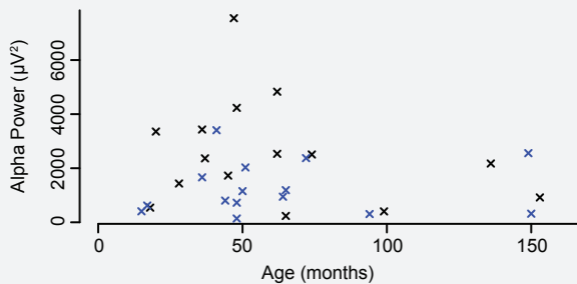

C

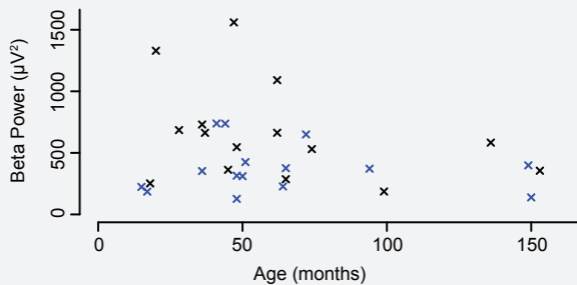

Supplement: Supplementary Fig. S1 — Relationship between patterns of brain activity with age. (A) The change in delta power in response to cannulation, (B) background alpha power, and (C) background beta power, plotted against age at study for term-born (black) and premature-born (blue) children. Each point represents the average EEG activity across channels for each child. [file mmc1.pdf]

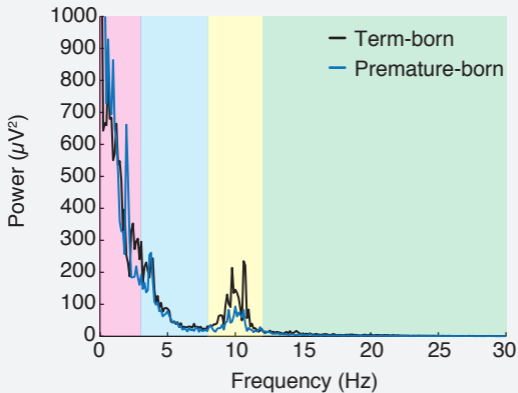

Supplement: Supplementary Fig. S2 — Average power spectra in the background EEG. Power spectra, averaged across all channels and all children, in the background period for the term-born (black) and premature-born (blue) children. For the analysis the power was calculated in the delta (0–3 Hz, red), theta (3–8 Hz, light blue), alpha (8–12 Hz, yellow), and beta (12–30 Hz, green) bands. [file mmc2.pdf]

**A**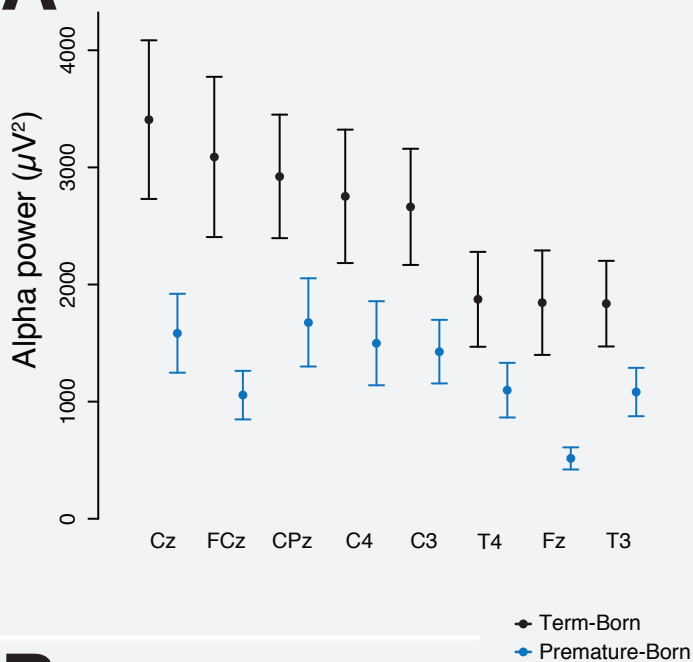**B**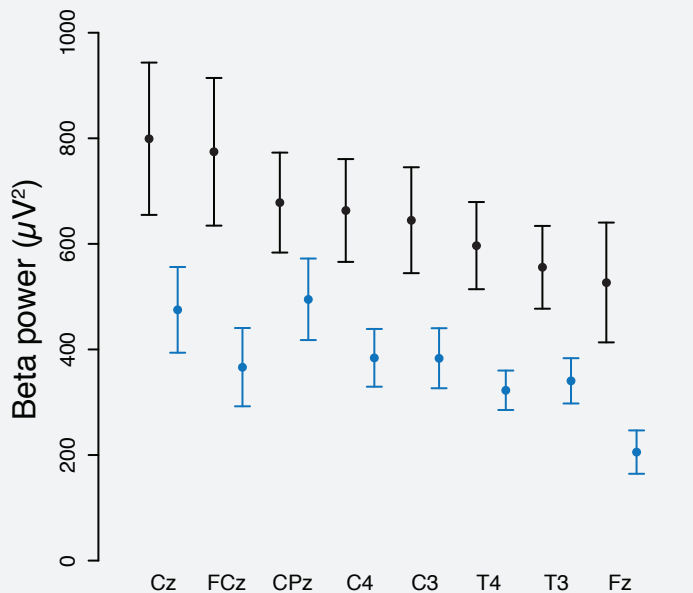

Supplement: Supplementary Fig. S3 — Comparison of band power with EEG electrode. (A) Alpha and (B) beta power in the first background period for each EEG electrode, compared between term-born (black) and premature-born (blue) children. Error bars indicate standard error of the mean. [file mmc3.pdf]

**A**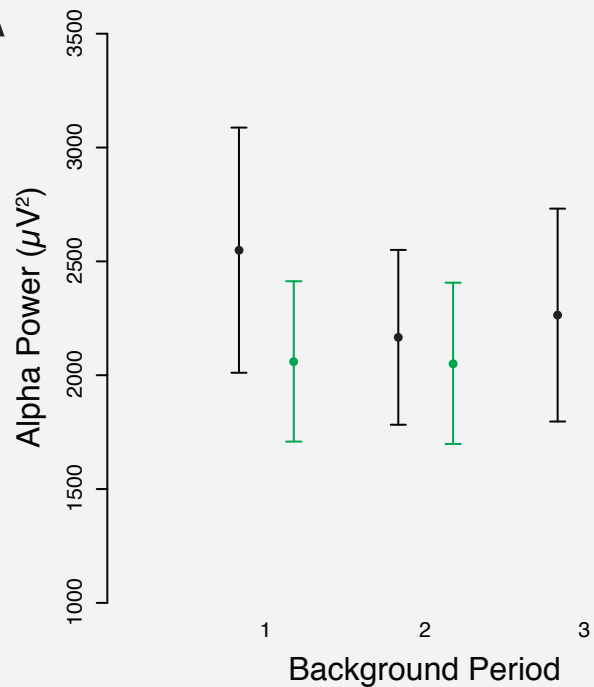**B**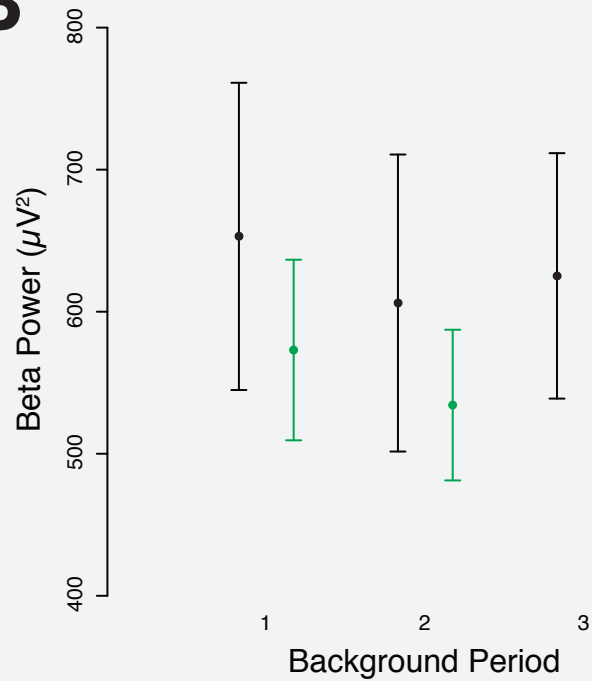

Supplement: Supplementary Fig. S4 — Comparison of band power in two groups of term-born children. (A) Alpha and (B) beta power across the three background periods in the group of term-born children studied in the main text (black) compared with a second group of term-born children (green). There were no significant differences between the two groups. Error bars indicate standard error of the mean. [file mmc4.pdf]
