# Supplementary material for: Electroencephalography during general anaesthesia differs between term-born and premature-born children
Source: Clin Neurophysiol. 2016 Feb;127(2):1216–22. doi: 10.1016/j.clinph.2015.10.041 (PMC4725254; doi:10.1016/j.clinph.2015.10.041)
Supplement: Supplementary Table S1 — Subject age and procedure details. Children are listed according to age, with age-matching indicated by each row of the table. Term-born children (blue rows) are divided according to whether they were in the main study (left hand side) or group 2 (right hand side). Details highlighted in red for the premature-born children (green rows) indicate those children with neurological impairment. [file mmc5.doc]

| **Term-Born** |  |  | **Premature-born** |  |  | **Group 2 Term-Born** |  |
| --- | --- | --- | --- | --- | --- | --- | --- |
| **Age (Months)** | **Procedure** | **Further details** | **Age (Months)** | **Procedure** | **Further details** | **Age (Months)** | **Procedure** |
| 18 | Orchidopexy |  | 15 | Retinal Detachment Surgery | IVH Grade 4 | 14 | Cystoscopy |
| 20 | Excision of Left Neck Mass |  | 17 | MRI Brain | Possible precocious puberty | 25 | Cystoscopy |
| 28 | CT Orbit | Possible dermoid cyst | 36 | Minor Eye Surgery |  | 32 | Trigger Thumb Release |
| 36 | MRI Brain | Vascular malformation over eye | 41 | Squint Repair |  | 33 | Orchidopexy |
| 37 | Manual Evacuation and Rectal Biopsy | Possible Hirschsprung's disease | 44 | Botox Injections | To treat hip displasia | 35 | Orchidopexy |
| 45 | MRI Brain | Stumbling frequently - MRI normal | 48 | Reduction Clitoroplasty | To treat clitoral hypertrophy | 39 | Squint Repair |
| 47 | Circumcision |  | 48 | MRI Brain | Bilateral Ventriculomegaly | 40 | Trigger Thumb Release |
| 48 | Hernia Repair |  | 50 | Squint Repair |  | 41 | Arthrogram |
| 62 | Orchidopexy and Preputial Adhesiolysis |  | 51 | Tonsillectomy and Adenoidectomy |  | 42 | Grommet Insertion |
| 62 | Hernia Repair |  | 64 | MRI Brain | Hydrocephalus | 62 | Arthrogram |
| 65 | Eye Examination and Refraction | Squint | 65 | MRI Brain | Spastic Dysplasia | 78 | Umbilicoplasty |
| 74 | Hernia Repair |  | 72 | Orchidopexy |  | 79 | Tibialis Anterior Transfer |
| 99 | Exploration of Perianal Fistula |  | 94 | Arthrogram |  | 83 | Squint Repair |
| 136 | Soft Tissue Release - Ankle |  | 149 | Multilevel Orthopaedic Surgery | Left Hemiplegia | 83 | Orchidopexy |
| 153 | Drainage of Humerus Osteomyelitis |  | 150 | Varus Osteotomies | Cerebral Palsy | 100 | Cystoscopy |
